# Persuasive technologies for modifying food habits: a review of mindless, reflective, and social approaches to eating behavior change

**DOI:** 10.3389/fdgth.2026.1750973

**Published:** 2026-03-10

**Authors:** Amon Rapp

**Affiliations:** Computer Science Department, University of Turin, Torino, Italy

**Keywords:** behavior change, eating, food, persuasive technologies, self-tracking

## Abstract

Eating habits are central to health and well-being, yet promoting lasting dietary change remains extremely challenging. In recent years, persuasive technologies have emerged as potential supports, leveraging personal data made available by mobile and wearable devices to provide targeted behavioral interventions. However, research in Human-Computer Interaction (HCI) highlights that these systems often fail to sustain people's engagement over time and may overlook the subjective, personal, and context-dependent nature of behavior change. Through an analysis of papers on persuasive technologies for healthy eating published at the ACM CHI Conference over the last ten years (2016–2025), this review maps the landscape of current digital instruments aimed at modifying people's eating habits, identifying several limitations: designs scarcely address the internal aspects of change, foster user agency, or account for the contextual and life factors that are central to behavior modification. In this sense, an alternative approach that values the subjective and existential aspects of the process of change could be explored in future research.

## 1 Introduction

There is broad agreement that eating habits play a crucial role in shaping people's health and well-being. Appropriate nutrition can help prevent chronic diseases and enhance overall quality of life (1). Yet, despite these positive outcomes, encouraging people to adopt a healthy diet remains extremely challenging, as many individuals do not possess a natural drive to eat well (2, 3).

In recent years, digital instruments have been proposed as a possible means of support. Persuasive technologies, specifically, are systems designed to influence users' behaviors (4), whose diffusion has been boosted by the widespread use of mobile and wearable devices (5–7). Weight-management apps, for instance, provide calorie monitoring, exercise routines, and community features (8, 9).

Despite their use having brought successes in modifying human behavior, Human-Computer Interaction (HCI) researchers have questioned whether such systems can create long-lasting changes (10–13). In fact, users often abandon these systems early, which significantly limits their potential benefits (14–18). A key limitation of current technologies is that they focus on quantifying eating behavior (e.g., calorie counts) and on externally driven strategies (e.g., points as incentives), while overlooking the subjective (19) and lived experience of behavior change (20). As also highlighted in prior work, the process of change as it unfolds in everyday life is often internally driven, shaped by personal meanings and motivations (20, 21).

In this article, I review 20 HCI papers on persuasive systems. As a first contribution, I provide a detailed picture of current persuasive designs in the food domain. As a second contribution, I offer a series of key points that potentially limit their effectiveness and that should be addressed by future persuasive designs.

## 2 A review of food behavior change systems

The HCI community has long investigated how technology can assist individuals in modifying their eating habits. To map the key themes addressed in this area, I analyzed 20 papers published in the ACM CHI Conference on Human Factors in Computing Systems Proceedings, the international leading conference in HCI. The choice of the CHI Proceedings ensures a focus on high-quality HCI works, while also limiting the scope of the review and the generalizability of its findings, as it excludes contributions from adjacent fields such as digital health and behavioral medicine.

I chose the ACM Digital Library as the source for searching articles, as it allows searches to be restricted exclusively to the CHI Proceedings. The search was conducted using the search terms (“behavior change” OR “behaviour change” OR “behavioral change” OR “behavioural change” OR persuas*) AND (food* OR eat* OR diet* OR weight). Retrieved articles were manually screened to assess whether they matched the inclusion criteria, first based on their titles and abstracts, and then by reading the full texts of the remaining works. Papers were included in the review if they appeared in the main CHI conference proceedings between 2016 and 2025; if they focused on eating behavior change; and if they introduced a system or analyzed the use of an existing technology aimed at influencing eating behavior. The analysis of the selected papers reveals three major research approaches: i) the mindless approach, where technology is used to subtly change people's behavior; ii) the reflective approach, where technology is aimed at increasing users' self-awareness; and iii) the social approach, where the social aspects of behavior change are addressed.

### 2.1 The mindless approach

A first strand of research tackled by the analyzed papers adopts a “mindless approach” to behavior change, which relies on the automatic, subconscious, processes driving people's behavior (22–24). This approach is mainly based on Applied Behavior Analysis (ABA) and behavioral economics, which either exploit environmental cues that may reinforce certain behavioral emissions (25), or introduce subtle changes in the way choices are presented with the goal of guiding users toward desired behaviors (26). For instance, systems grounded on ABA conceive behavior as the product of operant conditioning, namely, the pairing of a behavior with a reinforcement for increasing the future emissions of a wanted response (25). Behavioral economics, instead, relies on the notion of nudging, that is any aspect of the choice architecture that alters people's behavior in a predictable way: technologies based on this approach leverage people's heuristics (i.e., mental shortcuts) and biases (i.e., systematic deviations from rational judgment) to yield change in their behavior (26). Popular commercial mindless applications include, for example, HealthyWage^1^, which leverages loss aversion nudges (i.e., the fear of losing money), and Lose It!^2^, which uses default nudges by offering predefined diet plans.

In the reviewed articles, presented systems are mainly built on nudging mechanisms. For example, Kleinberger et al. (27) developed Auditory Seasoning, a mobile app that offers altered auditory feedback of eating sounds to influence food perception: experimental results highlight that amplifying eating sounds increases crispiness, as well as flavor-related parameters, like saltiness, and overall flavor intensity. Moreover, louder eating sounds may lead participants to feel fuller. Similarly, Q-Chef (28) uses surprising content to influence food decision-making. The authors found that surprising recipes evoke enthusiastic discussion of trying them. Along the same lines, DiCosola III & Neff (29) examine whether people's responses, during the checkout process of food product purchases, differ when their basket's calorie content is compared with that of other normal-weight adults (in-group comparison) or with that of overweight adults (out-group comparison). Their findings indicate that out-group comparison nudges can prompt users to opt for items with fewer calories.

In sum, mindless systems mostly employ narrowly defined intervention strategies that concentrate on the specific behavior to be modified. They may exploit subtle changes in the environment that alter people's perceptions or cognition and consequently unconsciously modify their behavior, or “social nudges” leveraging peer pressure and social comparison to induce change.

### 2.2 The reflective approach

A second approach emphasizes the role of reflection and rational processing as primary means to produce behavior change [e.g., (30–32)]. This approach builds on the increasing availability of behavioral information collected through self-tracking technologies: it assumes that people often lack insight into their own habits, and that acquiring such “self-knowledge” can lead to personal change (19). Li et al.'s (33, 34) personal informatics model, for example, which is widely used within the reflective approach, describes a sequence of stages that users move through when engaging with tracking tools. The process concludes with a reflection phase, in which individuals reflect on their data, followed by an action phase, where they modify their behavior based on this deeper understanding. Popular commercial product examples include MyFitnessPal^3^, which allows users to continuously track their eating habits, and Noom^4^, which uses mini-lessons to educate people about their food choices.

In the reviewed corpus, the main strand of research exploiting the reflective approach precisely leverages self-tracking technologies, with the aim of increasing people's self-awareness of their own eating habits. The analyzed papers focus on several key aspects of self-tracking practices. For instance, Chen et al. (35) show that context-sensitive prompts, like reminders for regular self-monitoring tailored to the user's current context, may encourage users to record their data. Karkar et al. (36), instead, tackle the problem of how to support self-experimentation, a prevalent practice among people who self-track to get concrete answers to specific questions. They present a mobile application that uses self-experimentation to help individuals with irritable bowel syndrome identify their personal food triggers.

More generally, Silva et al. (37) focus on the future opportunities that self-tracking technologies may open in the food domain. They carried out co-design workshops to envision how conversational interfaces might assist users with their food objectives. The outcomes of these workshops emphasize that people want self-tracking devices to adapt by learning their goals and preferences, to offer sufficiently detailed guidance that remains aligned with their aims, and to deliver feedback in an empathetic and non-judgmental manner. However, the reviewed literature also shows awareness of the limitations and potential drawbacks of these technologies. O’Neill et al. (38) observe that commercial food-tracking applications reinforce prevailing normativities surrounding diet, health, and the body, while failing to acknowledge the barriers that prevent access to the foods that are framed as “healthy”. They also promote the quantification of health, conveying the simplistic idea that the act of self-tracking alone may improve health.

A second strand of research embracing the reflective approach, instead, builds on the so-called “mindful eating”. Systems based on this perspective incorporate some principles of mindfulness practice and are designed to increase people's self-awareness and food awareness. ViFeed (39), for example, is a video playback system that uses subtle speed adjustments and glanceable visual cues to foster food awareness, food appreciation, and sustained engagement. Similarly, FoodCensor (40) creates moments of interruption that heighten users' awareness of how often they encounter digital food cues. Bomfim et al. (41) and Epstein et al. (42) present gamified systems aimed at improving individuals' food-related knowledge, providing them with challenges that may make them reflect on their food choices. Finally, GlucoGoalie (43) offers personalized nutrition goal suggestions for individuals with type 2 diabetes, which can subsequently be taken as an opportunity for reflection.

To summarize, the intended aim of all these systems is to help users build knowledge about food behaviors and encourage reflection. Nonetheless, they generally do little to support people in making sense of their own experiences or in developing new personal meanings around food. In this sense, they privilege the rational analysis of eating behavior over the interpretation of meaningful insights.

### 2.3 The social approach

A final approach identified in the reviewed articles involves the engagement of “others” in the process of change. Examples of commercial technology-based interventions include Weight Watchers^5^, which provides expert-led group coaching, and MyNetDiary^6^, which offers social features that allow users to create or join support groups. This approach highlights the importance of social aspects in shaping behavior and is mainly grounded in empirical studies that explore how people change, or are supported to change, in everyday life. In this sense, this line of research appears to take people's lived experience into greater consideration than the other two approaches.

A first line of research based on the social approach tackles the technology needs of people facing a behavioral change. Ha et al. (44), for example, point out that life changes like moving or unemployment may disrupt food routines and social dining. Current technologies often overlook these changes and do not sufficiently support people in collaborating with others to adapt to these transformations. The authors interviewed 18 participants who experienced routine changes during life events, finding that they need tools to facilitate social coordination, adapt to food practices, and mediate conflicts during transitions. Likewise, Barbarin et al. (45) discovered that obese or overweight women want to feel good about themselves and connected to other people, preferring emotional support from individuals who have similar experiences with overweight/obesity, are in the same phase of life, or share their social position. By contrast, Hentschel et al. (46) explore the cultural factors that may influence diet management in India. They show that food practices of people with diabetes are a collective effort, with the spouse, children, and neighbors contributing to such practices. In this context, culture may appear to subtly affect people's food choices, like nudges and reinforcements. However, the influence of culture appears to be far more effective in shaping people's eating habits, being embedded in people's values and beliefs, as well as in their social networks.

A second line of research concerns the study of professionals who provide support to individuals seeking to change their food habits. Rutjes et al. (47) emphasize that coaches value the experiential and relational aspects of coaching, which may serve a variety of goals, including understanding a client's implicit motivations, encouraging someone to overcome personal barriers, and providing adequate social support. Similarly, Ryan et al. (48) conducted interviews with eHealth coaches discovering that they try to adapt their support by tailoring educational content, setting individualized goals, and shaping their approach on the basis of the unique characteristics of their clients. Moreover, coaches strive to build the client-coach relationship as the foundation for providing such support.

Both of these research lines highlight the limitations of current systems in providing people with adequate social support for their behavior change attempts. In contrast, a third line of research examines how people appropriate existing technologies to obtain the kind of help that many persuasive systems fail to provide. Chung et al. (49) interviewed 16 women and explored how they use Instagram to keep a record of activities relevant to their eating goals: in doing so, they obtain and provide social support and information to communities with which they identify. Similarly, Chancellor et al. (50) analyzed online posts on two Reddit weight loss communities emphasizing that “norms matter” in how different communities provide support for health and well-being goals.

In sum, the papers addressing the social aspects of behavior change highlight that people need social support to carry out the change process, and that technology is not always able to provide it. However, individuals can also appropriate existing technologies to seek and offer help.

## 3 Discussion

Traditional behavior change methods often entail face-to-face sessions with trained practitioners and include assessing the client, setting goals, and providing guidance in using tools (e.g., tracking instruments) (48, 51, 52). In this context, technology may allow behavioral interventions to reach more people in efficient and cost-effective ways (48). For example, while self-monitoring can be difficult to sustain over long periods of time using traditional methods based on pen and paper (53, 54), technology offers instruments for real-time recording of food consumption and the automatic calculation of calorie intake (55). Moreover, technology enables just-in-time interventions that can be delivered anywhere, anytime (35, 56), whereas traditional methods might be confined to sessions with professionals. Nevertheless, the experience provided by technology might be less meaningful, and thus produce fewer positive outcomes than traditional interventions (47, 48).

In this review, I identified three different technology-based approaches. The social approach emphasizes the role of social support in behavior change and highlights that current technologies often fail to provide such support. The mindless and reflective approaches, instead, point out the opportunities that technology may offer. These two approaches appear to have brought successes in terms of behavior modification. Key positive achievements of mindless systems mainly revolve around the effective modification of specific behaviors, especially in the short term and for individuals who are not willing to change or who are not aware that such change is needed. Likewise, the reflective approach is often effective in making people more aware of their current behaviors, highlighting problematic habits, as well as increasing knowledge about the behavior to be modified and about possible strategies to address it. Table 1 provides an overview of the main strengths, theoretical limitations, and challenges of the approaches identified in this review.

**Table 1:** Strategies, strengths, theoretical limitations, and challenges of mindless, reflective, and social approaches.

Approaches	Strategies	Strengths	Theoretical limitations	Challenges
The mindless approach	It relies on behavioral reinforcements and nudges to shape behavior below the threshold of conscious awareness.	Mindless interventions may effectively modify people's perceptions about food (27, 28) and steer their actual behavior (29). The approach may be particularly effective for people who are not willing to change or are not aware that a change is needed.	Nudges and reinforcements may have only momentary effectiveness: people may relapse to unhealthy food practices when the intervention is removed (10).	It may be difficult for mindless technologies to define the correct timing for delivering nudges and reinforcements (10). Moreover, it could be challenging to develop long-lasting educational effects using only digital nudges and reinforcements (10).
The reflective approach	It relies on self-monitoring and mindful eating to increase people's self-awareness and knowledge.	Reflective interventions may increase people's food knowledge (41, 42) and awareness of unhealthy behaviors (39, 40). They may allow individuals to learn strategies for addressing their behavioral problems (36), through self-experimentation and self-reflection (43).	Self-monitoring may excessively quantify health (38), while increased self-awareness and self-knowledge may not necessarily lead to behavior change (38).	It may be difficult for self-tracking technologies to adapt to users and provide personalized and empathetic feedback (37). Similarly, it may be challenging to provide meaningful insights that can really increase users' self-awareness (38).
The social approach	It exploits “others” to deliver interventions based on personal bonds and social relationships.	The social approach primarily aims to provide social support (44–46). It may tailor the support individually to the unique needs, preferences, and goals of the user (47, 48), or exploit existing social media and online communities (49, 50).	“Others” may be unavailable, disengage, or inadvertently demotivate (57). Moreover, professionals delivering interventions might be difficult to reach (48). As social media are not designed to support healthy eating, they lack specific features to support behavior change (49).	It may be difficult to design technologies that provide deep connection with others (44, 45). It may also be challenging to balance social media norms (e.g., not to disappoint one's own followers) and someone's personal health goals (49).

On a closer look, however, the reflective and mindless approaches present even more substantial limitations (38, 47, 48).

Firstly, they ignore the wider life circumstances in which behavior change unfolds. Mindless technologies address only the target behavior to be changed, and although they can influence immediate actions, they often overlook the broader contextual factors in which the behavior occurs, like existing social relationships and everyday routines. Without considering the broader life context, just-in-time recommendations and reminders provided by these technologies may be perceived as annoying (58). The same holds true for the technologies grounded on the reflective approach. Despite promoting self-awareness and self-knowledge, such awareness and knowledge are often narrowly focused on the target behavior and do not concern the individual's “self” (19), or their lived experience (47). For instance, apps like Noom and MyFitnessPal place almost exclusive emphasis on calories, rather than on other more holistic life data. Nevertheless, previous research on food choices highlights that people's life course experiences generate major influences on dietary decisions: such influences include, for example, resources (e.g., monetary), social relationships, and the food context (e.g., the availability of foods in the food system) (59, 60).

Secondly, both approaches do not sufficiently consider the internal aspects of change, which involve the meanings, emotions, values, and motivations associated with food. For instance, many of the reviewed systems do not consider the subjective factors that may influence behavior “from the inside.” Moreover, the data visualizations provided by reflective apps using numbers and stats may not encourage the users' internal sense-making process (7), where their focus on numbers may produce anxiety and promote idealized body images (61). Nonetheless, prior research has emphasized the importance of such subjective factors in the process of change (20, 21, 47, 62).

Finally, the reviewed technologies appear to scarcely value the user's agency, often proposing solutions that steer behavior “from the outside.” For instance, nudges like preset meal plans may make people feel that their autonomy is taken away leading them to abandon the intervention (10). However, this also applies to technologies developed within the reflective approach. Self-tracking devices, for example, are often presented as tools that enhance user autonomy; however, they often also promote dependence on tracked data, ultimately reducing the individual's sense of agency (63, 64). Prior studies on self-regulation in eating behavior, however, point out that developing individuals' agency and self-control is important for the success of the intervention (51, 65, 66).

By contrast, even though commercial applications relying on social features may foster social comparison that discourages certain users (67) and promote diet norms that are not culturally appropriate for all users (50), the reviewed papers that deal with the social aspects of eating behavior seem to be more aware of the limits of current technologies. By emphasizing the importance of the social dimension in the process of change, like the relationship that can be developed between a coach and a client, they shift the focus from the behavior and the technology to the “existential aspects” of change. In this context, what really matters concerns the emotional support that other people can provide, the co-construction of meanings with other individuals, and the values shared by people within a culture. Table 2 offers a series of design examples coming from commercial products and research prototypes outside the CHI community, as well as design limitations and opportunities.

**Table 2:** Design examples, limitations, and opportunities of mindless, reflective, and social approaches.

Approaches	Research and product examples	Design limitations	Design opportunities
The mindless approach	As for research examples, in Forwood et al. (68) a grocery shopping website uses a “suggesting alternative” nudge (10), where for each food the system searches for a possible alternative and suggests the food swap. Guo and Wan (69) present Qingqing, a chatbot that, when tailored as a health coach, uses a priming nudge to encourage healthier food choices. As for commercial product examples, HealthyWage is based on a loss aversion nudge, by allowing users to bet on their weight loss: if they do not reach their target weight they lose the bet money. Lose It! uses default option nudges by suggesting predefined dieting plans and recommended calories per meal.	Just-in-time recommendations, reminders, or alternatives may be perceived as annoying or cause friction and reactance after repeated exposure (58). Nudges like default options used in Lose It! may make people feel that their autonomy is taken away leading them to abandon the intervention (70).	LLMs could deliver personalized nudges that may contextually steer users’ behavior: for example, they could generate just-in-time prompts based on users’ past data. Research has shown that personalizing digital nudges may increase their effectiveness (71). While nudging was initially conceived as a one-size-fits-all approach (10), LLMs provide new opportunities for tailoring nudges to particular contexts and users. Designers should tackle the ethical responsibility of designing digital nudges, especially those that limit users’ autonomy. For example, for the “default” nudge, designers should define how easily users can opt out and who bears the responsibility when an inappropriate default is offered and unwanted consequences arise (10).
The reflective approach	As for research examples, MyndFood is a conversational agent designed to promote mindful cooking and eating by encouraging users to pay attention to the textures, scents, and sounds of the cooking process (72). Foodbot utilizes a chatbot-based interface to record food intake and facilitate self-monitoring (73). As for commercial product examples, the MyFitnessPal app allows people to track their eating habits through barcode scanning which facilitates the self-monitoring process. Noom prompts users to complete mini-lessons to increase their nutritional knowledge.	The data displayed by reflective apps through charts, stats, and numbers may not be meaningful *per se*, as users may find it difficult to understand and make sense of the data visualizations: these may be too abstract and may not provide holistic views of the user's “self” (7). Apps like Noom and MyFitnessPal place a strong emphasis on calories and promote normative perspectives on health (e.g., by providing specific goals) (38). This may produce a fixation on numbers, increase users’ anxiety, foster app dependency, and promote idealized and unreachable body images (7, 38, 61).	Sense-making could be encouraged by chatbot-based LLMs, which could build data-driven “stories” that help users identify with their data. Alternatively, designers could provide more concrete and intuitive graphical representations, by using data-driven avatars that may change as the collected data change (17), or by relying on text-to-image models to automatically generate novel visualizations.
The social approach	As for research examples, SlimMe is an empathic diet chatbot acting as a friendly assistant to help users estimate their calorie intake and calories burned (74). REWIND is a virtual community-based weight management program that employs one-on-one virtual encounters, virtual forums, and team-building activities (75). As for commercial product examples, the Weight Watchers app provides expert-led group coaching where people may find advice and support. MyNetDiary offers the ability to create or join groups, where people may post their achievements using photos and find support from community members.	Virtual communities may entail social comparison risks, where seeing high-performing peers can produce lower self-evaluations due to perceived inferiority or increase shame or discouragement (67). Diet norms, food access, and body ideals vary widely and community support and advice provided by the social features of apps like MyNetDiary may not be culturally appropriate for all users (50).	LLM-based communities could provide multiple artificial agents that give support while showing empathy (76). They could also provide personalized advice tailored to the user's cultural background.

In this sense, a perspective that takes a different approach, foregrounding the individuals' lived experience of change may complement and amplify the impact of what has been achieved through the mindless and reflective approaches. Future HCI research should therefore devote greater attention to these internal and existential aspects of behavior change (21), also drawing inspiration from the social approach. With this aim, technology interventions could leverage novel conversational technologies based on Large Language Models (LLMs). These systems could emulate empathy and help individuals develop new meanings around the behavior to be changed. For example, virtual communities where multiple LLM-based artificial agents are present could provide always-available empathic support. However, since these technologies do not truly “understand” meanings and values, nor feel any emotions (77–79), their use in behavior change interventions should be approached with great caution. Moreover, LLMs could deliver more effective nudges, by tailoring them to the user's context and history, even though designers should take the ethical responsibility of their effects. Finally, LLMs and text-to-image models could help designers define new ways of displaying users' self-tracked data, by creating data-driven stories and images embedding users' data.

## Footnotes

^1^ https://www.healthywage.com/

^2^ https://www.loseit.com/

^3^ https://www.myfitnesspal.com/

^4^ https://www.noom.com/

^5^ https://www.weightwatchers.com/

^6^ https://www.mynetdiary.com/
